# A Hospital Impostor

**Published:** 1886-12-11

**Authors:** 


### A Hospital Impostor.

A Touching Story.

At a time like the present, when the pressure on the poor is increasingly great week by week, the detection and punishment of begging impostors—the worst enemies of the helpless—amounts to an urgent public duty. About half-past four on the afternoon of the 2nd inst., the private secretary of a gentleman who takes a special interest in the welfare and management of hospitals was passing into St. James's Street, from Park Place, when he was accosted by a respectable-looking artisan in his working costume, who expressed a desire to speak to him. The man said he had been for thirteen days out of employment, and had come to London five days before, from Bristol, in search of work. He had that day succeeded in obtaining employment in the Gas Department of the Board of Works, Westminster, and gave his name and address as George Roberts, 84, Latimer Road, Notting Hill. At his success in getting work he was rejoiced; but, unfortunately, that very morning his little girl, aged five years, had been taken suddenly ill, he thought, from want of proper food, and was in urgent need of hospital treatment. Could the gentleman, therefore, procure her admission to a hospital without delay?

Its Sequel.

Arrangements were made to investigate the case, and its *bona fide* character seemed to be confirmed from the fact that the man refused a shilling, as he said, "I am not begging. I really want help for my child"; though he added, "But I should be very grateful if you would lend me two shillings, which I will repay on Saturday week, to help me get some tools out of pawn." This request was complied with, and steps were immediately taken to secure the child's admission to a hospital. Alas, for poor human nature! When the gentleman drove to the address given early on the following morning, to take the child as a patient to St. Mary's Hospital, he found that George Roberts was not known at 84, Latimer Road, save only from the circumstance that another kind-hearted philanthropist had already been there on a similar errand of mercy, and with a similar result! Our correspondent says this is a particularly bad case, because the artisan looked so eminently respectable, and so thoroughly genuine. We publish this statement in the hope that this intelligent impostor may accost some other gentleman connected with a hospital, and that thus George Roberts may find his way into custody, and so be prevented from continuing his crusade against the best interests of the really deserving poor.

